# Cerebrospinal Fluid Concentrations of Biogenic Amines: Potential Biomarkers for Diagnosis of Bacterial and Viral Meningitis

**DOI:** 10.3390/pathogens7020039

**Published:** 2018-04-13

**Authors:** Aneela Taj, Nusrat Jamil

**Affiliations:** Department of Microbiology, University of Karachi, Karachi 75270, Pakistan; ain2005_ku@yahoo.com

**Keywords:** biogenic amines, bacterial meningitis, viral meningitis, cerebrospinal fluid, HPLC-EC, central nervous system, diagnosis

## Abstract

Catecholamine and serotonin are biogenic amines (BAs) that serve as neurotransmitters and play an important role in the regulation of cardinal functions that are mainly altered during central nervous system (CNS) infections. A total 92 samples of cerebrospinal fluid (CSF) were classified into 4 groups based on their etiology. In these samples, BAs/neurotransmitters i.e., dopamine (DA), 3,4-dihydroxyphenylacetic acid (DOPAC), homovanillic acid (HVA), and 5-hydroxyindoleacetic acid (5HIAA) were detected and quantified by high performance liquid chromatography with electrochemical detection (HPLC-EC) to determine the neurophysiology of the CNS infections by bacteria (*Listeria monocytogenes* (Lm) and *Neisseria meningitidis* (Nm)) and herpes simplex virus (HSV). CSF concentration of DA, DOPAC, HVA, and 5HIAA were found significantly elevated in all test cohorts. Present study highlights that the analysis of BAs is pivotal for the early diagnosis of bacterial and viral meningitis. In addition, coinfections of varied etiology can also be diagnosed by their quantification. Thus, BAs can serve as potential biomarkers of these CNS infections.

## 1. Introduction

Cerebrospinal fluid (CSF) is a watery liquid that circulates within the ventricles of the brain, and surrounds the brain and spinal column. It contains small molecules, salts, peptides, proteins, and enzymes that play critical roles in many physiological processes of the human host. Changes (concentration; modification of protein and peptides) in its composition reflect pathological processes in central nervous system (CNS) [1,2].

An extensive literature survey has revealed that a few studies have been reported in recent past that examined the concentration of amino acids in the CSF during bacterial [3,4,5,6,7,8] and viral meningitis [8]. Conversely, Molero-Luis et al. [9] has recently reported the abnormal values of homovanillic acid (HVA) i.e., a neurologically important intermediate product of dopamine degradation, in CSF of children suspected with neurological disorders.

Biogenic amines (BAs) refer to the neurotransmitters associated with several neurological and systemic functions governed by autonomic and central nervous system [9,10]. They are mainly involved in movement, sleep, learning, memory, thermoregulation, emotional behavior, as well as other functions [10,11]. BAs are extensively studied to understand pathogenesis of neuronal injury induced by a variety of CNS insults of neurological, physical, biochemical, and physiological origin [9]. However, quantification of BAs during CNS infections caused by bacterial and viral etiological agents has not yet been studied.

Therefore, interest in the quantification of BAs is stimulated by an attempt to explore their role in the active state of CNS infection of varied etiology. Consequently, this study will contribute additional evidence to understanding the pathogenesis of these infections. The present study was designed to quantify the concentration of BAs in the CSF samples resulted in the isolation of bacterial and viral etiology.

## 2. Results

### 2.1. Quantification of Dopamine (DA)

This experiment was delineated to quantify the concentration of BAs in infected CSF samples. Concentrations of biogenic amine (BA) neurotransmitters were quantified in all groups i.e., Nm, Lm, Nm+HSV, and HSV by taking standard mean. Diagnostic analysis of CSF is usually recommended by the doctors of patients with mental ailments which could be infectious or physiological disorders. A healthy individual can never go through the trauma of the procedures of the CSF collection. Therefore, hypothetical composition of the CSF is available but the details of the healthy personnel CSF are scarcely available in the literature. Keeping these facts in mind, intra group comparative analysis was done in the present study. However, the baseline of BAs was determined by the external standards.

**Figure 1:**
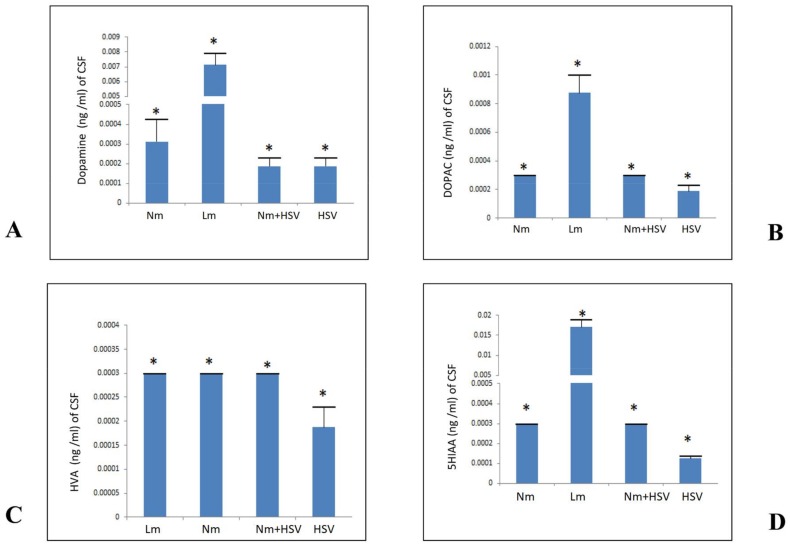
Quantification of biogenic amines in CSF samples showed varied etiologies for CNS infections. (**A**) Concentration of dopamine; (**B**) concentration of DOPAC; (**C**) concentration of HVA; and (**D**) concentration of 5HIAA. All data presented herein subsequent to relative analysis within intra patient groups. Values are expressed as mean ± SEMs * (*p* < 0.05).

It was significant to note that, among these four cohorts, the highest level of dopamine (DA) was found (*p* < 0.05) in the Lm cohort (72.5 ng/mL of CSF) (Figure 1A). However, the Nm group revealed a lower amount of this BA than the Lm group quantity (1.875 ng/mL of CSF). Whereas the same quantity of DA (in ng/mL of CSF) was found in the CSF samples infected either solely with HSV (1.25 ng/mL of CSF) or coinfection of Nm+HSV (1.25 ng/mL of CSF).

### 2.2. Quantification of 3,4-Dihydroxyphenylacetic Acid (DOPAC)

Another set of experiments was conducted to quantify the DA degradation intermediate i.e., 3,4-dihydroxyphenyl acetic acid (DOPAC). It is worth mentioning here that, in the Lm group the relative quantity of DOPAC (0.181 ng/mL of CSF) was found to be significantly elevated compared to the rest of the cohorts (*p* < 0.05) (Figure 1B). Although in this, the precursor i.e., DA was found to be 70× greater than the rest of the cohorts. However, relatively lowest quantity (0.022 ng/mL of CSF) of DOPAC was detected in the CSF of HSV group. Conversely, Nm and Nm+HSV groups revealed relatively same quantity of this BA i.e., (0.035 ng/mL of CSF).

### 2.3. Quantification of Homovanillic Acid (HVA)

The downstream degrading product of DOPAC i.e., homovanillic acid (HVA) was detected in all four CSF groups infected with varied etiologies. The relatively highest quantity of this BA degradation pathway intermediate was found to be 0.027 ng/mL of CSF of Nm and Nm+HSV cohorts. Two-way ANOVA showed a significant relative reduction (*p* < 0.05) in the quantity of HVA in Lm (0.023 ng/mL of CSF) (Figure 1C). Whereas intra group comparison (*p* < 0.05) revealed that the lowest quantity (0.0136 ng/mL of CSF) of this BA was found in HSV cohort.

### 2.4. Quantification of 5-Hydroxyindoleacetic Acid (5HIAA)

In this set of experiment, 5-hydroxyindol acetic acid (5HIAA) levels were quantified in CSF during the active state of infection. It is significant to note that relatively highest quantity (*p* < 0.05) of 5HIAA (1.365 ng/mL of CSF) was found in Lm group (Figure 1D). However, the relatively lowest quantity of 5HIAA (0.100 ng/mL of CSF) was yielded by the HSV group. Furthermore, intra group comparison (*p* < 0.05) revealed that Nm and Nm+HSV groups showed relatively same levels (0.300 ng/mL of CSF) of this BA.

Finally, Lm infection stands out with the elevated biomarkers i.e., DA and 5HIAA.

## 3. Discussion

The conventional laboratory examination of cerebrospinal fluid (CSF) for the diagnosis and treatment of various central nervous system (CNS) infections consists of CSF pressure, measurement, total and differential leukocyte count, glucose and protein quantification, Gram stain, bacterial and viral culture, and polymerase chain reaction (PCR) of different etiological agents. Conversely, we focused on exploring the significance of the quantification of biogenic amines (BAs) in the CSF which may additionally contribute in the existing diagnostic tools of CNS infections.

It is worth mentioning herein that analysis for BAs in CSF samples reflected a correlation between varied concentrations of these with infection associated etiology (bacteria/virus). Present study emphasized that infections may disturb the neurotransmitters degradation pathway. In the present study, comparative analysis of the results has indicated complete downstream degradation of dopamine (DA) into its metabolic products i.e., 3,4-Dihydroxyphenylacetic acid (DOPAC) and Homovanillic acid (HVA) in HSV infection whereas, in Lm infection, DA catabolized into DOPAC devoid of further degradation into HVA (Figure 1A–C). Although there is documented fact in literature that elevated concentrations of neurotransmitters in CSF confirm the disruption of blood brain barrier which was reported to be significant early pathophysiologic change in meningitis [12,13]. However, results of the present study take these findings a step further and describe that neuropathogens are competent enough to alter the neurophysiology of the host, thus significantly contributing to understanding the new venue of pathogenesis of CNS infection.

**Figure 2:**
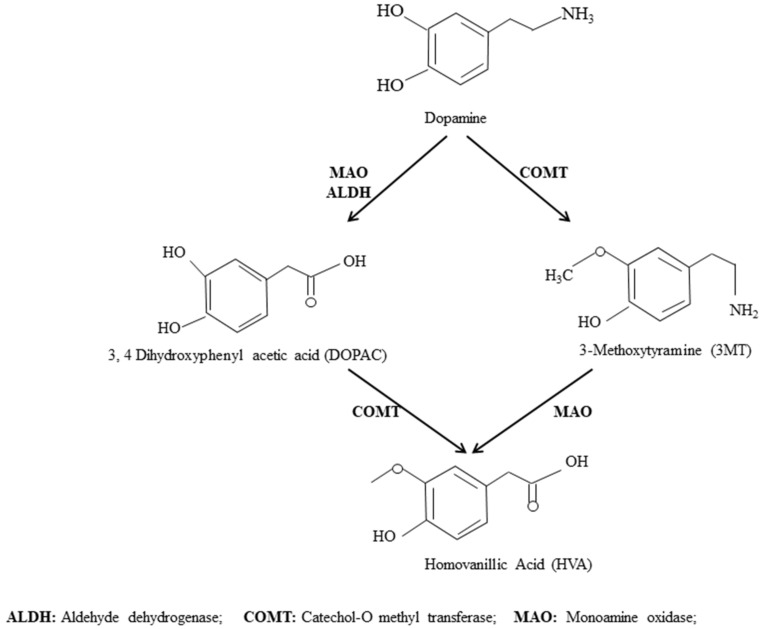
Schematic representation of the dopamine degradation pathway.

Transitionary elevation of DA in response to pain, stress, and fear is a well-documented fact. However, sustained elevation in its concentration is attributed to pathological manifestations in response to any etiological agent. Conventionally, DA is released into the synaptic cleft as a consequence of excitation of presynaptic dopaminergic neurons where it interacts with the DA receptors of postsynaptic neuron and initiates signaling cascades such as Na^+^/K^+^ channels for the generation of action potential. Elimination of extracellular DA from the synaptic cleft is mandatory and it occurs either when DA gets recycled after reuptake by dopaminergic neurons or degraded after uptake by glial cells [14,15] by following the conventional pathway (Figure 2). Contrary to this, results of the present study reflected the elevated quantity of DA (72.5 ng/mL of CSF) (Figure 1A) and reduced concentration of HVA (0.023 ng/mL of CSF) (Figure 1C) in the Lm group which highlights that this bacterium can alter the metabolic pathways of DA. The results of the present study are in line and can be further strengthened by the findings of another study performed in our lab in which BA neurotransmitters were quantified in the whole brain of Sprague Dawley rat models subsequent to the injection of sterilized cell free broths (SCFBs) of Lm [16]. Interestingly, a significant increase in the DA level (5057 ng/g of brain) and a reduction in HVA level (2.5 ng/g of brain) in the Lm cohort superimpose the results of the present study. Bacterial SCFBs mimic the infection scenario, therefore present study suggested that the enhanced concentration of DA and a comparatively low concentration of its downstream products in CSF samples and in animal models reflected the intervention in metabolic pathways of DA by the Lm. Limitations of our lab facility did not allow us to work with HSV in animal models. The study was confined only to CSF analysis of BAs.

**Figure 3:**
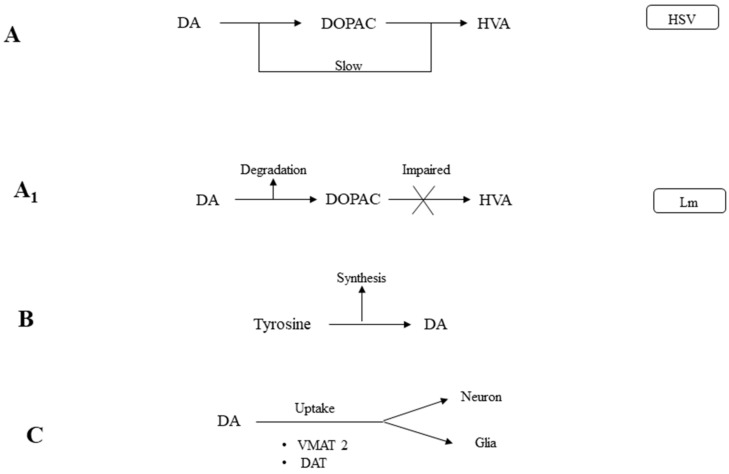
Hypothesized models of altered biogenic amine levels during bacterial and viral meningitis.

The results of the present study can be further explained by the schematic models A and A1 (Figure 3) that indicated metabolic intervention i.e., in A. The degradation machinery seems to be impaired as it could happen in HSV infections. The relative concentration of DA and DOPAC and the relatively lower amount of HVA indicated the malfunction of the relevant enzymes. Whereas model A1 proposes a high rate of synthesis of DA as compared to its further degradation into products. The absence of HVA indicated the complete inhibition of catechol-O methyl transferase (COMT) by Lm. Model B illustrates the increased synthesis of DA via its precursor amino acid. The other possible explanation for significantly enhanced concentration of DA is the impaired activity of uptake system for DA that vesicular monoamine transporter (VMAT) and dopamine transporter (DAT) responsible to transport DA into glial and neuronal cells. Therefore, DA accumulated in the synaptic cleft or in the cytoplasm.

In a nutshell, our study obviously indicated the variation in the pattern of neurotransmitter degradation in bacterial and viral infections. The increased quantity of DA in Lm the animal cohort [16] is superimposed with a high yield of DA in CSF of Lm infection which prompts us to propose that DA biochemistry is affected by Lm infection. Therefore, it can be concluded that neuropathogens and the extracellular metabolites released by them during their growth, facilitate disease progression in the nervous system and brain. Even if etiological agents remain localized in body parts other than the brain, its metabolites i.e., Lipopolysaccharide, Peptidoglycan, and/or peptides are potent enough to disturb the biochemistry and physiology of the CNS.

## 4. Materials and Methods

### 4.1. Chemicals

Unless otherwise stated, biogenic amine standards were obtained from Sigma, St. Louis, MO, USA. High performance liquid chromatography with an electrochemical detection (HPLC-EC) system was acquired from Merck, Frankfurter, Darmstadt, Germany with Chromeleon v.6.8.0 software.

### 4.2. Cerebrospinal Fluid (CSF) Samples

The present study is a sub-analysis of cerebrospinal fluid (CSF) samples collected randomly from patients admitted with neurological ailments in the two major government hospitals of Karachi, Pakistan [17]. Details of patients’ symptoms are summarized in (Table 1). Collection of the CSF samples was carried out with the consent and approval of lab authorities. All persons gave their informed consent prior to inclusion in this study. All the collected CSF samples were aliquotted and stored at −80 °C immediately.

All procedures performed in this study involving human participants were in accordance with the 1964 Helsinki declaration and its later amendments.

### 4.3. Study Groups

All the CSF samples were screened for the detection of the bacterial and herpes viral etiological agents. Viral detection was carried out by two different set of specific primers targeting glycoprotein G genes through PCR as described earlier [18]. Whereas bacterial identification was carried out by standard conventional methods i.e., culture, microscopy and biochemical tests as previously described [17]. Consequently four groups of CSF samples were classified. Group 1 and 2 were comprised of CSF samples that exclusively yielded bacterial infection i.e., *Neisseria meningitidis* (Nm) and *Listeria monocytogenes* (Lm) respectively. While group 3 was composed of CSF samples showed bacterial and HSV coinfection (Nm+HSV). Furthermore, group 4 was consisted of CSF samples yielded the presence of HSV strains i.e., (HSV1) and Herpes simplex virus 2 (HSV2) (HSV) (Table 2).

CSF samples those proven the presence of infection either with single etiology or coinfection with varied (bacterial and viral) etiological agents were selected and used for conducting these experiments. To analyze the presence of biogenic amines, representative CSF samples were taken from each of the four groups (Table 2). Groups 1, 3 and 4 were consisted of 2 CSF sample/group whereas group 2 was comprised of 1 CSF sample/group. Furthermore, CSF samples that failed to yield the presence of any etiology were excluded from present study.

### 4.4. Chromatographic Conditions

HPLC-EC system was consisted of a Dionex ED50 electrochemical detector, a glassy carbon working electrode, 5 μm Octadecyl silane (ODS) reverse phase, 250 × 4.6 mm, C-18 column and Chromeleon v.6.8.0 software. The column buffer consisted of 0.1 M NaH_2_PO_4_, 0.1% octyl sodium sulphate, 0.0035% EDTA, 14% methanol (pH 2.9 adjusted with phosphoric acid). The flow rate of the pump (Dionex Ultimate 3000) was 1 mL/min. The sensitivity of the detector was 1.0 nA and potential of the working electrode was 0.6 V. 25 μL of each CSF sample was injected into the HPLC-EC system. The peak areas generated from the CSF samples were recorded and quantified by comparison with external standards containing 100 ng/mL of dopamine (DA), 3,4-dihydroxyphenylacetic acid (DOPAC), homovanillic acid (HVA), and 5-hydroxyindoleacetic acid (5HIAA). All of these experimental procedures were run in triplicate.

### 4.5. Statistics

The significance (*p* < 0.05) of between group differences was analyzed using the two-way ANOVA tool in SPSS version 15. Statistical data are expressed as mean ± SEMs.

## 5. Conclusions

CSF’s DA and/or its downstream conversion products seem to be good biomarkers for identifying the ongoing bacterial and/or viral infection. Therefore, we strongly recommend the detection of biogenic amines for thorough diagnosis of neuro-infections.

## Figures and Tables

**Table 1:** Description of the patients’ symptoms along with the infection.

Infection	Symptoms
*Neisseria meningitidis* (Nm)	High fever
	Headache
	Vomiting
	Fits
*Listeria monocytogenes* (Lm)	High fever
	Headache
*Neisseria meningitidis* +Herpes simplex virus (Nm+HSV)	High Fever
	Headache
	Unconsciousness
Herpes simplex virus (HSV)	Fever
	Fits
	Severe headache
	Low vision

**Table 2:** Description of the experimental groups used in the current study.

Study Groups	Infection Type	Etiologies	Detection Method	Reference
Group 1	Single	*Neisseria meningitidis* (Nm)	Bacterial Culture	[17]
Group 2	Single	*Listeria monocytogenes* (Lm)	Bacterial Culture	[17]
Group 3	Coinfection	Nm + Herpes simplex virus (Nm+HSV)	Bacterial Culture PCR	[17,19] [18,19]
Group 4	Single	Herpes simplex virus (HSV)	PCR	[18,20]
